# The Microbiome of the Reef Macroalga *Sargassum ilicifolium* in Singapore

**DOI:** 10.3390/microorganisms9050898

**Published:** 2021-04-22

**Authors:** Ren Min Oh, Elena Bollati, Prasha Maithani, Danwei Huang, Benjamin J. Wainwright

**Affiliations:** 1 Department of Biological Sciences, National University of Singapore, 16 Science Drive 4, Singapore 117558, Singapore; dbsohrm@nus.edu.sg (R.M.O.); e.bollati@nus.edu.sg (E.B.); prashamaithani@u.nus.edu (P.M.); huangdanwei@nus.edu.sg (D.H.); 2 Centre for Nature-Based Climate Solutions, National University of Singapore, 16 Science Drive 4, Singapore 117558, Singapore; 3 Tropical Marine Science Institute, National University of Singapore, 18 Kent Ridge Road, Singapore 119227, Singapore; 4 Yale-NUS College, National University of Singapore, 16 College Avenue West, Singapore 138527, Singapore

**Keywords:** bacterial communities, macroalgae, marine microbiome, microbial ecology, plant–microbe interactions, Southeast Asia

## Abstract

The large canopy-forming macroalga, *Sargassum ilicifolium*, provides shelter and food for numerous coral reef species, but it can also be detrimental at high abundances where it outcompetes other benthic organisms for light and space. Here, we investigate the microbial communities associated with *S. ilicifolium* in Singapore, where it is an abundant and important member of coral reef communities. We collected eight complete *S. ilicifolium* thalli from eight island locations along an approximate 14 km east-to-west transect. Each thallus was dissected into three separate parts: holdfast, vesicles, and leaves. We then characterized the bacterial communities associated with each part via polymerase chain reaction (PCR) amplification of the 16S rRNA gene V4 region. We then inferred predicted metagenome functions using METAGENassist. Despite the comparatively short distances between sample sites, we show significant differences in microbial community composition, with communities further differentiated by part sampled. Holdfast, vesicles and leaves all harbor distinct microbial communities. Functional predictions reveal some separation between holdfast and leaf communities, with higher representation of sulphur cycling taxa in the holdfast and higher representation of nitrogen cycling taxa in the leaves. This study provides valuable baseline data that can be used to monitor microbial change, and helps lay the foundation upon which we can begin to understand the complexities of reef-associated microbial communities and the roles they play in the functioning and diversity of marine ecosystems.

## 1. Introduction

The macroalga, *Sargassum ilicifolium* (Phaeophyceae: Sargassaceae), is a large and abundant canopy-forming brown seaweed found throughout the coral reefs of Singapore. It provides habitat and food for numerous reef-associated species [1,2], and at high abundances it can be a competitor with other benthic organisms for light and space [3]. Microbial associations with algae are ubiquitous in marine systems [4,5,6,7,8,9]. These associations help improve host fitness and in return the host provides habitat, carbohydrates and other nutrients along with oxygen that can be used by bacteria [10]. Bacteria are able to promote algal growth, prevent the fouling of photosynthetic surfaces by epiphytes, and play crucial roles in nitrogen cycling, metabolism and fixation [11,12,13].

*Sargassum ilicifolium* dominates many reef flats in Singapore where it comes into frequent contact with corals [1,14]. This contact can be detrimental to benthic organisms via indirect mechanisms, such as smothering, abrasion, shading [15,16], and direct mechanisms, including allelopathy [17,18,19]. The presence of macroalgae can reduce the survivorship of marine larvae, which in turn limits settlement and consequently recruitment, thereby inhibiting the potential of degraded reefs to recover [16]. Similarly, coral–macroalgae interactions can alter the coral microbiome [20], which is essential to the coral host health [21,22,23,24]. Notably, coral–macroalgae interactions on degraded reefs are hypothesized to drive dysbiosis of the coral microbiome [25,26], and macroalgae have been implicated as a source of harmful microbes that reduce the fitness of corals [27,28].

Research examining the macrophyte-associated microbiome shows that microbial communities associated with different tissues or plant structures contain different communities, likely a consequence of the different roles different parts play in promoting host survival [29,30]. These communities can also be significantly different even over relatively small spatial scales, such as less than 10 km in some cases [31,32,33,34]. Photophysiological and biochemical heterogeneity between structures has been documented for *Sargassum* spp., with differences in photosynthetic rates [35], phlorotannin content [36] and C:N ratios [37] being reported. The chemical microenvironment found in different structures could thus play a role in shaping microbial community composition and regulating host–microbe interactions. In seagrasses, distinct microbial communities are associated with blades, roots and rhizomes, where they play key roles in the carbon, nitrogen and sulphur cycle at both the holobiont and ecosystem level [29]. Similarly, we expect to observe community differences in the different tissues of *Sargassum ilicifolium* (e.g., the leaf-like laminae will harbor different communities in comparison to the vesicles or the holdfast).

There is rapidly increasing interest in the marine microbiome, particularly in how coral reefs and other coastal organisms such as mangroves and seagrasses interact with one another, and more broadly regarding how the entire marine microbiome can be influenced by the species present along with terrestrial and atmospheric inputs and interactions [38]. Considering the high abundance of *Sargassum ilicifolium* in the waters of Singapore, the acknowledged influence the microbiome has on host survival, and the growing interest in how species will interact and change under the projected climate change scenarios, characterizing microbial communities in marine environments is important. It provides a baseline on which we can monitor change and, importantly, it provides a framework on which we can build to understand the complex interactions that constitute the marine microbiome as a whole.

## 2. Materials and Methods

**Figure 1:**
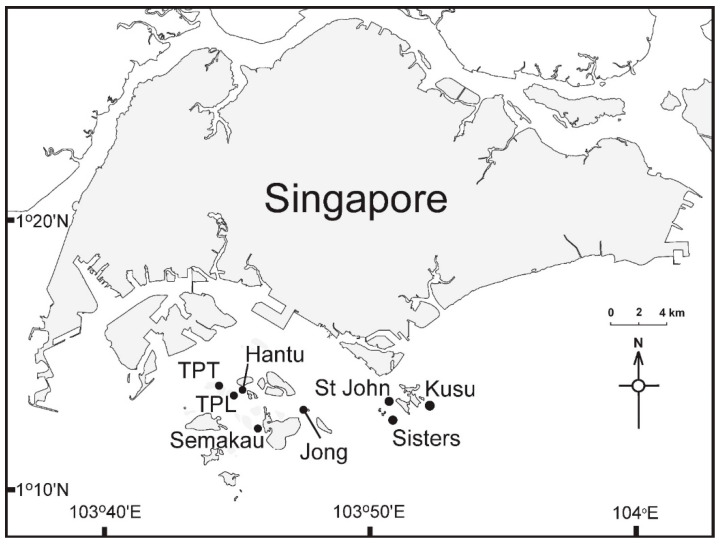
Map of sample locations throughout Singapore.

Samples were collected and DNA extracted as described in [39]. Briefly, eight entire *Sargassum ilicifolium* thalli were collected over two days in January 2018 from each of the eight islands (Figure 1). All thalli appeared healthy (e.g., no visible signs of disease, uniform coloration and free from epiphytes). Individual thalli were then separated into leaves, holdfast and vesicles. All samples were then surface-sterilized by immersion in 1% NaClO solution prepared with DNA-free autoclaved water for 2 min, 70% EtOH for 2 min and rinsed twice in sterile, DNA-free autoclaved water for 5 min. Surface sterilization is a common and effective technique used to prevent the polymerase chain reaction (PCR) amplification of DNA that is found on the surface of tissues [39,40,41,42,43]. It is considered the gold standard [44] and the application of bleach NaClO ensures DNA is denatured [45,46].

All tissues were disrupted in an Omni Bead Ruptor 24 (Omni International) at 8 m/s for 2 min, and DNA was extracted with a Qiagen DNeasy Powersoil kit following the manufacturer’s instructions. PCR amplification targeting the V4 region of the 16S rRNA gene was performed using the bacterial and archaeal primers 515F and 806R (515F—GTG CCA GCM GCC GCG GTA A; 806R—GGA CTA CHV GGG TWT CTA AT). Forward and reverse primers were modified to include Illumina adaptors, a linker and a unique barcode [47]. Each reaction was performed in a total volume of 25 µL, containing 1 µL of undiluted template, 0.1 µL of KAPA 3G Enzyme (Kapa Biosystems, Inc, Wilmington, MA, USA), 0.75 µL of each primer at 10 µM, 12.5 µL KAPA PCR Buffer and water to 25 µL. the PCR cycling protocol was 94 °C for 180 s, followed by 35 cycles of 94 °C for 45 s, 50 °C for 60 s and 72 °C for 90 s, with a final extension at 72 °C for 10 min. Negative extraction and PCR controls were included to identify possible contamination issues.

PCR products were visualized on a 1% TBE buffer agarose gel. The normalization and cleaning of PCR products were performed in SequalPrep normalization plates (Invitrogen, Frederick, MD, USA) and submitted for sequencing on the Illumina MiSeq platform (600 cycles, V3 chemistry, 300-bp paired end reads) with a 30% PhiX spike (Macrogen Korea).

Sequences were demultiplexed, and barcodes and adaptors removed with Cutadapt [48]. Reads were filtered based on quality scores and trimmed using the DADA2 package version 1.14.1 [49] in R version 3.6.2. Forward reads were truncated at 260 bp, and reverse reads were truncated at 160 bp. Both forward and reverse reads were filtered to remove any reads with a max expected error (EE) of 2, and reads were additionally truncated at the end of “a good quality sequence” with the parameter truncQ = 2 (see https://benjjneb.github.io/dada2/ for a detailed explanation of filtering parameters, accessed on 22 April 2021).

The DADA2 algorithm was used to estimate error rates from all quality-filtered reads and to merge forward and reverse reads and infer amplicon sequence variants (ASVs). Chimeras were removed with de novo detection. Sequenced extraction negatives were used to identify and remove any possible contaminants using the prevalence method implemented in the decontam R package [50], and remaining ASVs were assigned taxonomy with the RDP classifier [51] against a training set based on the Silva v138 16S database [52].

Any ASVs assigned to mitochondrial or chloroplast genomes and those not present in at least 5% of samples were removed. Rarefaction curves were produced using the rarecurve() function implemented in the vegan R package version 2.5.6 [53]. Sequences were aligned with MAFFT v7.429 under --auto setting [54] before phylogenetic inference with the FastTree plugin on QIIME2 [55,56]. Using the “Phangorn” package [57] in R, trees were mid-point rooted and weighted UniFrac distances were calculated. Community dissimilarity was subsequently visualized on principal coordinate analysis (PCoA) plots. Permutational multivariate analysis of variance (PERMANOVA) was performed on wUniFrac measures with the adonis function (number of permutations = 10,000) in the “vegan” R package in order to test for community differences among algal structure and island locations. Raw sequence counts were converted to relative abundance. Plots of relative abundance were made using ggplot2 version 3.3.1 [58] and Mann–Whitney U tests were performed to determine whether significant differences in diversity exist.

The filtered ASV table was used to predict metagenome functions using METAGENassist [59]. ASVs with the same taxonomic assignment were collapsed and remaining variables were filtered based on interquartile range; the data were then normalized across samples by sum and across variables by autoscaling (mean-centered and divided by variable standard deviation). Data were analyzed by phenotype “metabolism” and visualized by PCA.

All sequences associated with this work have been deposited at the National Center for Biotechnology Information under BioProject ID: PRJNA681720.

## 3. Results

**Figure 2:**
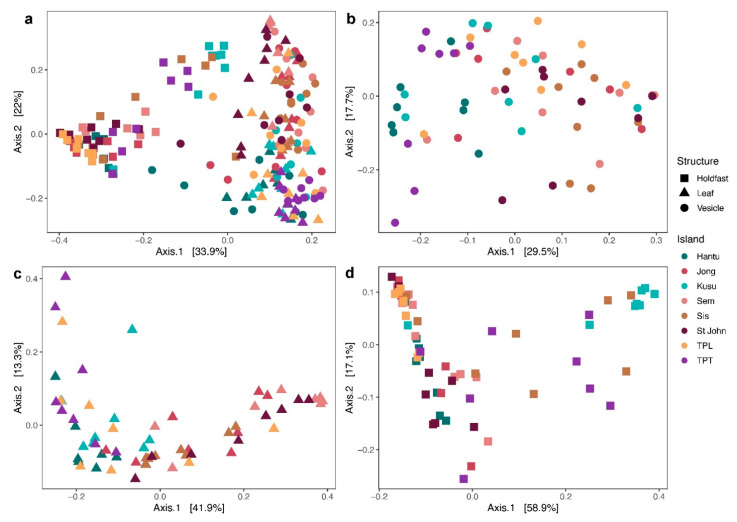
Weighted UniFrac principal coordinate analysis (PCoA) of microbial communities. Analysis plots of (**a**) all structures from all locations combined, (**b**) vesicles from all locations, (**c**) leaves from all locations, and (**d**) holdfasts from all locations.

A total of 14,683,748 sequences were generated on the Illumina MiSeq platform. After filtering to remove chimeric sequences and any sequences that did not pass quality control, a total of 11,343,322 sequences were retained for analysis (for sequencing statistics of each sample, see Table S1). Rarefaction curves show that all samples were sequenced to sufficient depth to achieve asymptote, indicating that all diversity was recovered (Figure S1). The PCoA plots for the combined data across all structures show that microbial communities associated with the holdfast are distinct from leaves and vesicles, while plots by individual structure suggest weak differentiation by island locations generally (Figure 2). These patterns are supported by the results of PermANOVA that indicate significant differences in microbial community composition between structures (*R*^2^ = 0.30, *p* < 0.001) and between island locations (*R*^2^ = 0.14, *p* < 0.001) (Table S2). There was a significant interaction between structure and island location as well, suggesting that the differences in microbial communities between locations are distinct among structures.

**Figure 3:**
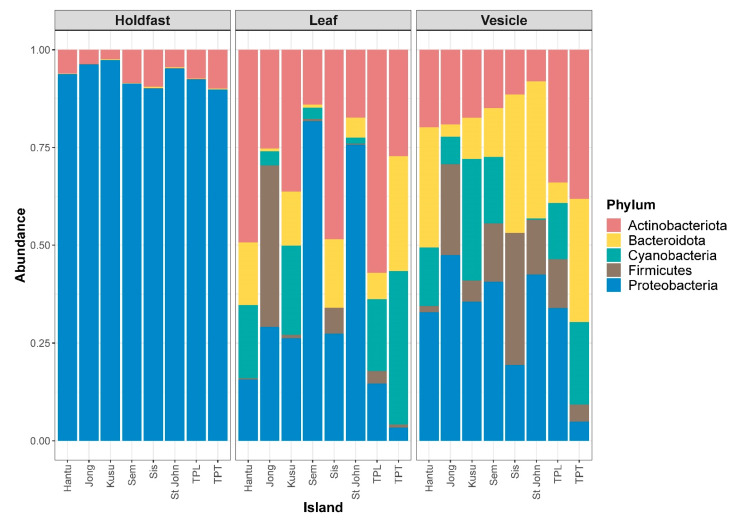
Stacked bar plot of the top 20 amplicon sequence variants (ASVs) at the phylum level for each structure characterized.

**Figure 4:**
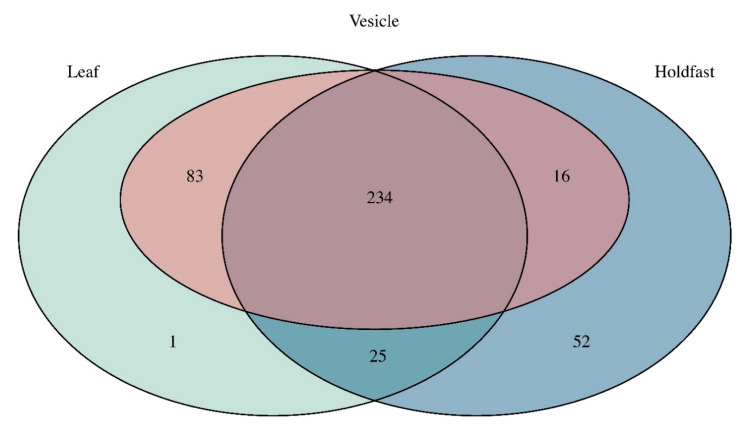
Venn diagram showing the number of ASVs unique to and shared between each structure.

Barplots of diversity show that the holdfast is most distinct in terms of community composition when compared to the leaves and vesicles. The holdfast is dominated by the phylum Proteobacteria, while leaves and vesicles have a more even composition of Actinobacteriota, Bacteroidota, Cyanobacteria, Firmicutes, and Proteobacteria (Figure 3). The uniqueness of the holdfast remains at the levels of class, order, and family, whereas the leaves and vesicles have communities that are similar in composition (Figures S2–S4). Similarly, the Venn diagram shows that the holdfast has the highest number of unique ASVs of all three structures, whereas the highest number of shared ASVs is found between the leaves and vesicles at 83 ASVs; in total, 234 ASVs are shared between all three structures (Figure 4). Plots of diversity show that leaves and vesicles contain a similar level of diversity and are not significantly different from each other (*p* > 0.05), while the holdfast is significantly different from the leaves and vesicles (*p* < 0.01) and contains the lowest diversity. Diversity at all islands is similar and not significantly different between any pairwise comparisons (*p* > 0.05) (Figures S5 and S6; see Table S3 for diversity metrics of each individual sample and Table S4 for details of ASV abundance and assigned taxonomy).

The metagenome functional predictions showed considerable overlap in metabolism-related phenotypes between communities sequenced from holdfasts, leaves and vesicles (Figure S7a). The loading plots showed that some separation between holdfast and leaf communities was associated with the higher representation of sulphur cycling taxa (sulphur oxidizer, sulphur metabolizing) in the holdfast, and the higher representation of nitrogen cycling taxa (nitrogen fixation, nitrite reducer) in the leaves (Figure S7b).

## 4. Discussion

In this study, we use high-throughput amplicon sequencing to characterize the microbial communities associated with the commonly encountered reef macroalga, *Sargassum ilicifolium*, from eight island locations in Singapore along an approximate 14 km east-to-west transect. We show that microbial communities are significantly different between locations despite the short distances separating them, and communities can be further differentiated dependent upon the structure sampled (e.g., leaf, vesicle or holdfast), with holdfasts appearing to have the most distinct community composition. These findings are in agreement with other work from the region that shows microbial community divergence at small spatial scales in a diverse range of marine taxa such as seagrasses [33], mangroves [32] and corals [60].

There are no obvious barriers to dispersal between the islands in this study, and the prevailing water currents run from east to west with semi-diurnal tidal oscillations [61]. These oceanographic conditions should facilitate host microbial community homogeneity, particularly over the small spatial distances between sample sites. The waters surrounding the islands in this study are well mixed [61] and can be considered homogeneous in terms of physical characteristics with comparable water quality throughout the Singapore Strait year round [62,63]. These stable conditions have resulted in fish and benthic assemblages that are very similar throughout the Strait [64], yet despite the similarities in macrofaunal composition we show that microbial communities are significantly different over the same spatial scales.

The differences seen in the microbial communities associated with *S. ilicifolium* from different locations are not a consequence of seasonality, as all collections were made over a two-day period in January 2018. Rather, we suggest, much like *P. acuta* [24], the significant differences in microbial communities from different locations are a result of environmental heterogeneity. For example, Terumbu Pempang Laut (TPL) and Terumbu Pempang Tengah (TPT) are submerged reefs, while all other locations sampled are adjacent to islands that are permanently above the highest spring tides. All sample sites are uninhabited, but the islands do receive occasional visitors to varying degrees. Semakau is the site of Singapore’s only offshore landfill disposal facility, and St John’s Island is home to the St John’s Island National Marine Laboratory and other facilities that host a daily work force. It is conceivable that the characteristics of each sample location (e.g., fully submerged reef vs. island and the degree of associated anthropogenic disturbance) could be influencing community structure [25]. It is also possible that the fine-scale physical properties of the water column surrounding each sample site are influenced by the surrounding substrate—island or submerged reef. Islands will produce higher levels of more localised freshwater runoff in comparison to the submerged reef sample sites, and the characteristics of this effect are determined by the island size, degree of vegetation and the activities that take place on each island. For example, St John’s Island—with the marine research lab, various aquaculture facilities and numerous day trippers—would have a distinct runoff composition compared to the Sisters’ Islands, which are part of the protected Sisters’ Islands Marine Park.

Localized rainfall-created runoff does not affect the submerged reefs in this study; while salinity will be reduced in the short term in the waters over these reefs, it will quickly return to the same salinity as the surrounding water with tidal mixing. Conversely, precipitation that falls on islands requires time to reach the reef; while this means that drops in salinity are likely more gradual and less dramatic, they probably occur over a longer duration and take on the chemical characteristics of the substrate they travel over or through (e.g., changes in pH, or elevated nutrient loads and increased sedimentation associated with vegetation and other human activities that take place on each) [65,66]. Our present work does not explicitly test these ideas, and further studies investigating the effects of precipitation runoff on water quality in reefs are required, but changes in salinity, nutrient loading and increased disturbance are known to alter microbial community structure [67,68,69,70]. It is likely that similar mechanisms are, at least partially, contributing to the differences in community structure we observe between sample sites.

Our analysis shows a differentiation in the microbial communities associated with the structures sampled, with the holdfast harboring the most distinct community. This is in line with studies performed on other marine organisms from the region, which have shown microbial community divergence over small spatial scales in seagrasses, mangroves and corals [32,33,34]; the same *S. ilicifolium* structures have also been shown to harbor distinct fungal communities in Singapore [39]. Like other studies examining algal associated microbiota, we show that holdfasts and leaves contain a large proportion of Alphaproteobacteria and Gammaproteobacteria; however, noticeable by their absence in both of these structures, samples collected in Singapore do not contain the class Flavobacteria in comparison to those collected from temperate environments [6].

Metagenome functional predictions suggest that leaf-associated communities contain more taxa with the potential to perform nitrogen cycle functions, while the holdfast-associated communities contain more taxa with potential sulphur cycling roles, and these predictions are consistent with results from *Sargassum horneri* collected in the Yellow Sea indicating that bacteria likely play important roles in nutrient cycling in Sargassum species [7]. Nitrogen fixation has been documented as a source of new nitrogen in *Sargassum* communities, particularly under nutrient limitation [70,71], and endophytic diazotrophs have been reported from anoxic microenvironments in other macroalgae [72]. Sulphur cycling pathways on the other hand are commonly enriched in marine sediment in the presence of decomposing seaweeds or seagrasses [73,74], and this could potentially extend to the holdfast-associated communities. While this approach only takes into account functional pathways that have been previously described in the identified taxa and does not provide direct information on which pathways are actually being expressed in the community sampled, it nevertheless provides an indication of the genetic potential of the community to express certain functional pathways, and can thus serve as an informative tool to help formulate hypotheses and guide future studies. However, in order to account for transcriptional plasticity and horizontal gene transfer, a metatranscriptomic or metagenomic approach is recommended for future investigations into the roles of distinct *Sargassum*-associated microbial communities in nutrient cycling on coral reefs.

Our study characterizes the microbial communities associated with the reef macroalga, *Sargassum ilicifolium*, in the highly urbanized marine environment of Singapore. Characterizing the microbes present in marine environments, living within hosts, in the reef matrix, or water column, is an important first step in understanding how the marine microbiome is structured and could respond to change, and this is especially crucial for some of the most commonly encountered species such as *S. ilicifolium*. Work such as this provides foundational information that can be further advanced with the development and testing of explicit hypotheses that facilitate a greater understanding of how species and their associated microorganisms interact and evolve as environments change.

## Data Availability

All sequences associated with this work have been deposited at the National Center for Biotechnology Information under BioProject ID: PRJNA681720.

## Supplementary Materials

The following are available online at https://www.mdpi.com/article/10.3390/microorganisms9050898/s1, Figure S1: Rarefaction curves indicating sufficient sequencing depth was achieved to recover ASV diversity in each sample. Figure S2: Stacked bar plots of relative microbial abundance at the level of class. Figure S3: Stacked bar plots of relative microbial abundance at the level of order. Figure S4: Stacked bar plots of relative microbial abundance at the level of family. Figure S5: Plots of Shannon diversity by structure. Figure S6: Plots of Shannon diversity by sampled location. Figure S7: PCA (**a**) and loadings plot (**b**) of predicted metabolic phenotypes. Ellipses show 95% confidence intervals. Table S1: Sequencing summary statistics after filtering and quality control. See https://benjjneb.github.io/dada2/tutorial.html for a complete description of parameters, specifically the track reads through pipeline section. Table S2: PermANOVA showing that bacterial and archaeal communities are significantly different between sampled islands, and plant parts. Table S3: Shannon and InvSimpson diversity metrics for each individual sample. Table S4: Details of ASVs, assigned taxonomy and occurrence in each sample.
